# Females exhibit higher GluA2 levels and outperform males in active place avoidance despite increased amyloid plaques in TgF344-Alzheimer’s rats

**DOI:** 10.1038/s41598-022-23801-w

**Published:** 2022-11-09

**Authors:** Osama Chaudry, Kelechi Ndukwe, Lei Xie, Maria Figueiredo-Pereira, Peter Serrano, Patricia Rockwell

**Affiliations:** 1 Department of Biological Sciences, Hunter College CUNY, New York, NY USA; 2 PhD Program in Neuroscience, The Graduate Center CUNY, New York, NY USA; 3 Department of Computer Sciences, Hunter College CUNY, New York, NY USA; 4 Department of Psychology, Hunter College CUNY, New York, NY USA

**Keywords:** Neuroscience, Diseases

## Abstract

Alzheimer’s disease (AD) is a progressive neurodegenerative disease that is most prevalent in females. While estrogen provides neuroprotection in females, sex mediated differences in the development of AD pathology are not fully elucidated. Therefore, comparing events between sexes in early-stage AD pathology may reveal more effective therapeutic targets of intervention. To address sex differences, we analyzed early-stage 9-month male and female TgF344-AD (Tg-AD) rats, an AD model carrying the APPswe and Presenilin 1 (PS1ΔE9) mutations that develops progressive age-dependent AD pathology similar to humans. Tg-AD females significantly outperformed Tg-AD males in the active place avoidance (aPAT) test that assesses hippocampal-dependent spatial learning and memory. However, comparisons between Tg-AD male or female rats and their WT counterparts showed significant deficits for female but not male rats. Nevertheless, Tg-AD females experienced significantly less hippocampal neuronal loss with higher GluA2 subunit levels than Tg-AD males. Unexpectedly, Tg-AD females displayed higher levels of hippocampal amyloid plaques than Tg-AD males. Thus, we propose that GluA2 may provide a neuroprotective function for Tg-AD females in our rat model by mitigating cognitive impairment independently of amyloid plaques. Elucidating this protective mechanism in AD could lead to new targets for early intervention.

## Introduction

Alzheimer’s disease (AD) is a neurodegenerative disease that is the major cause of dementia in the US. Historically, women at 60 years show a greater risk than men of developing AD^1^. Some biological mechanisms that may increase the risk and progression of AD in women include changes in brain structure, stress, pregnancy, menopause, and sex hormones, the genetic trait APOE, inflammation, and vascular disorders^2^. There is a critical need to understand mechanisms that contribute to the greater risk for women to develop AD.

AD is characterized by the following hallmarks: cognitive deficits, extracellular amyloid beta (Aβ) plaques, intracellular neurofibrillary tangles, neuroinflammation, and neurodegeneration. In the current study we focus on all of these except neurofibrillary tangles, as they appear later in disease progression. AD pathology starts decades before the onset of clinical symptoms, with the entorhinal cortex and hippocampus as brain regions affected initially and most extensively^3^. The disease progresses from early to late stages following the classical trisynaptic pathway from entorhinal cortex (layer II) → dentate gyrus → CA3 → CA1^3^ which involves new memory acquisition and is vulnerable to premature degeneration^3^.

New evidence demonstrated that dysfunction in the GABAergic system, the major inhibitory neurotransmitter system in the CNS contributes to cognitive deficits in AD. GABA plays a central role in regulating neuronal signaling in the hippocampus that affects memory and cognition^4^. This dysfunction involves a neuronal excitation/inhibition (E/I) imbalance which occurs early in AD and further promotes AD pathogenesis^5^. Furthermore, decreased activity of GABA inhibitory interneurons results in structural and functional impairments of nerve circuits that contribute to cognitive deficits in AD^6^.

Other insults are related to circulating glucocorticoids acting through hippocampal receptors^7,8^, synaptic dysfunction and oxidative stress^9,10^. Elucidating the relationship between hippocampal plasticity/vulnerability and AD pathology in early AD could lead to new targets for early intervention.

Neurodegeneration and neuronal loss are features associated with amyloid plaque formation^11^ involving dysregulated proteolytic processing of the amyloid precursor protein (APP)^12^. APP cleavage by α-secretase within the Aβ domain produces sAPPα and an αCTF fragment which is further cleaved by γ-secretase^13^. In AD, APP processing is associated with cleavage by BACE1, a major β-secretase^14^ that produces a βCTF fragment and sAPPβ, followed by γ-secretase cleavage of the βCTF fragment to produce Aβ, yielding Aβ-40 and Aβ-42. Aβ dyshomeostasis is proposed as a major neurotoxic event leading to neurodegeneration in AD^15^.

AD also is linked to neuroinflammation based on post-mortem brain tissue of AD patients^16^. In AD, an imbalance of pro-inflammatory and anti-inflammatory signaling, in part due to microglia-released cytokines^17^, allows inflammation to become chronic^18^. Microglia normally remain in a surveillance state, and have small soma and long processes^19^. A threat to the CNS causes microglia to undergo a morphological change, triggering the release of pro-inflammatory cytokines^20^. In AD, it is hypothesized that Aβ activates microglia, which localize to plaques to phagocytose them^21^. Eventually, microglia are unable to clear the plaques, leading to prolonged inflammation, neuronal damage, and exacerbated AD pathology^22–24^.

To address potential sex differences in the early stages of AD pathology, we chose 9-month male and female TgF344-AD (Tg-AD) rats. This transgenic rat model of AD develops amyloid plaques, gliosis, neurofibrillary tangles, neuronal loss and learning deficits in an age-dependent manner spanning from 6 to 26 months of age^25^. The Tg-AD rat model expresses two human genes driven by the mouse prion promoter, APPswe (Swedish) mutation, and human presenilin-1 exon 9 deletion (PS1ΔE9)^25,26^ at levels that are respectively 2.6 and 6.2 higher than their endogenous rat counterparts^25^. The APPswe mutation promotes cleavage of APP by BACE1, leading to Aβ-40/42 deposition, and formation of Aβ oligomers and plaques in the hippocampus and cortex of Tg-AD rats^25,27^. The PS1ΔE9 mutation shifts Aβ generation to longer and more aggregation-prone Aβ peptides via changes to the γ-secretase complex^28^. The Tg-AD rat model is unique as it develops the full array of AD pathology in a progressive and age-dependent manner, thus mimicking disease progression in humans^25,29^.

Our studies focused on gaining a clearer understanding of the greater prevalence of AD in females compared to males by investigating events that develop differently between sexes in the early-stage of AD pathology. We identified significant sex differences in that 9-month Tg-AD females outperformed Tg-AD males in cognitive assessment but exhibited higher levels of hippocampal amyloid plaques and amoeboid microglia. Females also experienced less hippocampal neuronal loss and had higher GluA2 subunit levels than Tg-AD males, suggesting that GluA2 may play a neuroprotective role in maintaining cognition during early-stage AD. Elucidating this potential GluA2-dependent protective mechanism in AD could lead to new targets for early intervention.

## Materials and methods

### TgF-344AD transgenic rat model of AD

Fisher transgenic F344-AD (Tg-AD) rats^25^ expressing human Swedish amyloid precursor protein (APPswe) and Δ exon 9 presenelin-1 (PS1ΔE9) along with wild-type (WT) littermates were purchased from the Rat Resource and Research Center (RRRC, Columbia, MO), and arrived at Hunter College when they were approximately 4 weeks of age. The rats were housed in pairs on a 12 h light/dark cycle with food and water available ad libitum and maintained at the Hunter College Animal Facility. All experiments were performed in compliance with the regulations of the Institutional Animal Care and Use Committee (IACUC) at Hunter College. All experimental procedures were approved by the IACUC and were in agreement with the standards outlined in the ARRIVE guidelines.

### Experimental design

We included female and male rats at the age of 9-months in the following numbers: Tg-AD females n = 17, WT females n = 12, Tg-AD males n = 23, WT males n = 21. Hippocampal-dependent cognitive deficits were assessed with the active-place avoidance task (aPAT). Following behavioral testing, a subset of rats (Tg-AD females n = 6, WT females n = 4, Tg-males n = 10, WT males n = 5) were sacrificed, and the brains were rapidly isolated and bisected into hemispheres, and processed for the different assays as described below.

### Active place avoidance task (aPAT)

To access hippocampal dependent spatial working-memory we evaluated cohorts of rats on the active place avoidance task^30^, aPAT (Bio-Signal Group, Acton, MA) as previously described in^31^. The aPAT is an automated system that tracks the animals’ movements on a circular rotating platform. Rats received a shock (0.2 amps), when they enter the stationary shock zone. Using the room cues, the rats learn the location of the shock. Prior to training each rat received a 10 min habituation trial allowing animals to acclimate to the training environment. For each training day, subjects from every condition were evaluated. Each animal was given six 10 min trials with a 10 min, inter-trial interval. 24 h after the last training trial, the animal’s ability to avoid the shock zone in the absence of shock was evaluated. The system software recorded data for all trials, and all data was exported to .tbl files and analyzed offline (TrackAnalysis, Bio-Signal Group). In rare instances, animals are dropped from the study for failure to move or for repeated jump-escapes from the arena. There were no animals dropped from this study.

### Brain tissue preparation and immunohistochemical analysis (IHC)

At 9-months of age, the rats were anesthetized (i.p.) with ketamine (100 mg/kg) and xylazine (5 mg/kg) and transcardially perfused for 15 min. with cold RNAse-free PBS. The rat brains were removed, and the left hemisphere was micro-dissected (regions: prefrontal cortex, cingulate cortex, entorhinal cortex, and hippocampus) and immediately snap frozen for molecular or biochemical analyses. The right hemispheres were processed for IHC analyses. Brain processing and IHC were performed as described in^31^.

Briefly, the right hemispheres were sequentially post-fixed with 4% paraformaldehyde for 48 h at 4 °C and cryoprotected in a 30% sucrose/PBS solution at 4 °C until they sank to the bottom of the vial. They were then flash frozen in 2-methylbutane, and stored at − 80 °C until sectioned. The right hemispheres were sectioned with a Leica CM 3050S cryostat. Hippocampal coronal sections of 30 µm in thickness were collected serially along the anteroposterior axis and stored at − 20 °C in cryoprotectant [30% glycerol (Fisher BioReagents, cat# 15514029) and ethylene glycol (Fisher BioReagents, cat# 10532595)] in PBS until use.

IHC was restricted to dorsal hippocampal tissue within the following Bregma coordinates: − 3.36 to − 4.36 mm^32^. Sections were processed with a mounted protocol for IHC analyses as described in^33^. Briefly, hippocampal sections were washed in 1X PBS for 5 min. three times, followed by mounting and quenching with 0.05 M glycine (Fisher BioReagents, cat# BP3815) solution for 30 min. Sections were washed with 1X PBS/Triton X-100 0.3% (Life Technologies, Thermo Fisher Sci., cat# PI85112) three times for 5 min. and then blocked for 30-min. with 30% normal goat serum (Vector Labs, cat# S-1000) and 1X PBS/Triton 0.3%. Two to three sections (averaged) from each rat were immunostained for Aβ plaques, microglia (Iba1 antibody), mature neurons (NeuN antibody), GluA2 subunit, and PSD95. Following the overnight incubation with the primary antibodies, sections were washed three times for 5 min. with 1X PBS/Triton 0.03% and blocked for 15 min. in 30% NGS and 1X PBS/Triton 0.3%. After blocking, sections were incubated for 60 min. in the fluorescent secondary antibodies. Primary and secondary antibodies are listed in Supplemental Table 1. All antibodies were diluted in 30% NGS and 1X PBS/Triton 0.03%. Two sets of three washes at 5 min. each were performed after the secondary antibody incubation, with 1X PBS/Triton 0.03% and 1X PBS, respectively. Mounting media with DAPI (VectaShield, cat# sku H-1200-10) was used to mount, and sections were stored in the dark at 4 °C.

Primary and secondary antibodies are listed in Supplemental Table 2. For IHC and quantification two to three slices were used per rat. Aβ plaques (Fig. 2, IHC) and Aβ levels (Fig. 6, westerns) were assessed in rat hippocampal tissue with two different mouse monoclonal antibodies: 4G8 (Biolegend, cat #800708, Aβ aa 17–24) and 6E10 (Biolegend, cat #SIG-39320, Aβ aa 1–16 and full-length APP). The 4G8 antibody should recognize only Aβ, so we used it for IHC to detect plaques. The 4G8 antibody has a greater affinity for human Aβ (manufacturer’s specifications). The 6E10 antibody recognizes APP and many of its abnormally processed forms as well as precursor forms, so we used it for western blotting to distinguish APP and its different cleavage products including Aβ. The 6E10 antibody has a threefold higher affinity for human APP and Aβ compared to the rat counterparts (manufacturer’s specifications).

### Image processing and quantification

Sections were viewed on a Zeiss Axio Imager M2 microscope with AxioVision 4 module MosaiX software to capture ZVI files of 10× mosaic images of the whole hippocampus using a Zeiss AxioCam MRm Rev. 3 camera connected to a motorized stage. Signal density (O.D.) was quantified using Image J as previously described in^31^.

FIJI-ImageJ was used for all image adjustments and for quantification of microglia and plaque size, count, and percentage area in the cornu ammonis 1 (CA1), cornu ammonis 3 (CA3), dentate gyrus (DG) and subiculum (SB) hippocampal regions as described in^31^. To ensure equivalent quantification across all conditions, we included slices from each group whenever a batch of tissue was quantified. For IHC and quantification two to three slices were used per rat. To insure equivalent quantification across all conditions, we included slices from each group whenever a batch of tissue was quantified.

Each channel was analyzed to an antibody specific threshold. A GFP spectrum filter set was used for Iba1 imaging, a DSRed set was used Aβ imaging, and a DAPI set was used for DAPI imaging. Exposure time for each channel was kept consistent between sections. Each channel was analyzed to an antibody specific threshold. A GFP spectrum filter set was used for Iba1 imaging, a DSRed set was used for Aβ imaging, and a DAPI set was used for DAPI imaging. Exposure time for each channel was kept consistent between sections. For each captured image, ZVI files were loaded onto FIJI (Fiji Is Just ImageJ, NIH, Bethesda, MD) and converted to .tiff files for analyses. Images were analyzed to extract the positive signal from each image with custom batch‐processing macro scripts created for each channel/marker using the following formulae: average pixel intensity + [(1.5 [Iba1], 4.0 [Aβ], or (1[GluA2 and NeuN]) × Standard deviation of intensities]. FIJI-ImageJ was used for all image adjustments and for quantification of microglia and plaque size, count, and percentage area in the cornu ammonis 1 (CA1), cornu ammonis 3 (CA3), dentate gyrus (DG) and subiculum (SB) hippocampal regions.

### Microglia analysis

Microglia exhibit a variety of morphologies that associate with their functions and according to their form factor (FF) defined as 4π × area/perimeter^2^, s are distributed into three different groups^34,35^. Each group is defined as follows: *Ramified*, FF: 0 to 0.49; which actively engage in neuronal maintenance by providing neurotrophic factors, *Reactive*, FF: 0.50 to 0.69; which are responsive to CNS injury, and *amoeboid*, FF 0.70 to 1; which are amorphous with pseudopodia. Microglia within each cropped Iba1 image were extracted using the following formula: average pixel intensity + [1.5 × standard deviation of intensities], and particles within 50–800 µm^2^ were chosen for FF analyses. Nonspecific background density was corrected using ImageJ rolling-ball method^36^.

### Western blot analysis

Hippocampal tissue (20–25 mg) was homogenized in TBS containing protease and phosphatase inhibitors for 90 s at 25 °C with the Bedbug microtube homogenizer (3400 rpm, model D1030, Benchmark Scientific). The supernatant was stored for 16 h at − 80 °C, followed by centrifugation at 14,000 rpm for 20 min at 4 °C. We adopted this protocol to insure complete lysis of the homogenate. The supernatant was filtered using biomasher homogenizer tubes (#09-A10-050, OMNI International). Samples were stored at − 80 °C until use. Protein concentration was determined with the BCA assay (Pierce Biotechnology), followed by normalization. 30 μg from each sample were run on 4–12% SDS gels and transferred to nitrocellulose membranes with the iBlot^®^ dry blotting system (Life Technologies) for 7 min). Membranes were blocked with SuperBlock (#37535, ThermoFisher), and hybridized with various primary antibodies followed by HRP-conjugated secondary antibodies (Supplemental Table 2), prior to developing with an enhanced chemiluminescence (ECL) substrate (SuperSignal™ West Pico PLUS, ThermoFisher #34580), and detected on a BX810 autoradiography film (Midwest Scientific). ImageJ software (Rasband, W.S., ImageJ, U. S. National Institutes of Health, Bethesda, Maryland, USA, https://imagej.nih.gov/ij/, 1997–2018) was used for semi-quantification by densitometry of the respective bands. Loading controls used were GAPDH or β-actin depending on their molecular weights to avoid overlapping with the other proteins studied.

### Statistics

**Figure 1:**
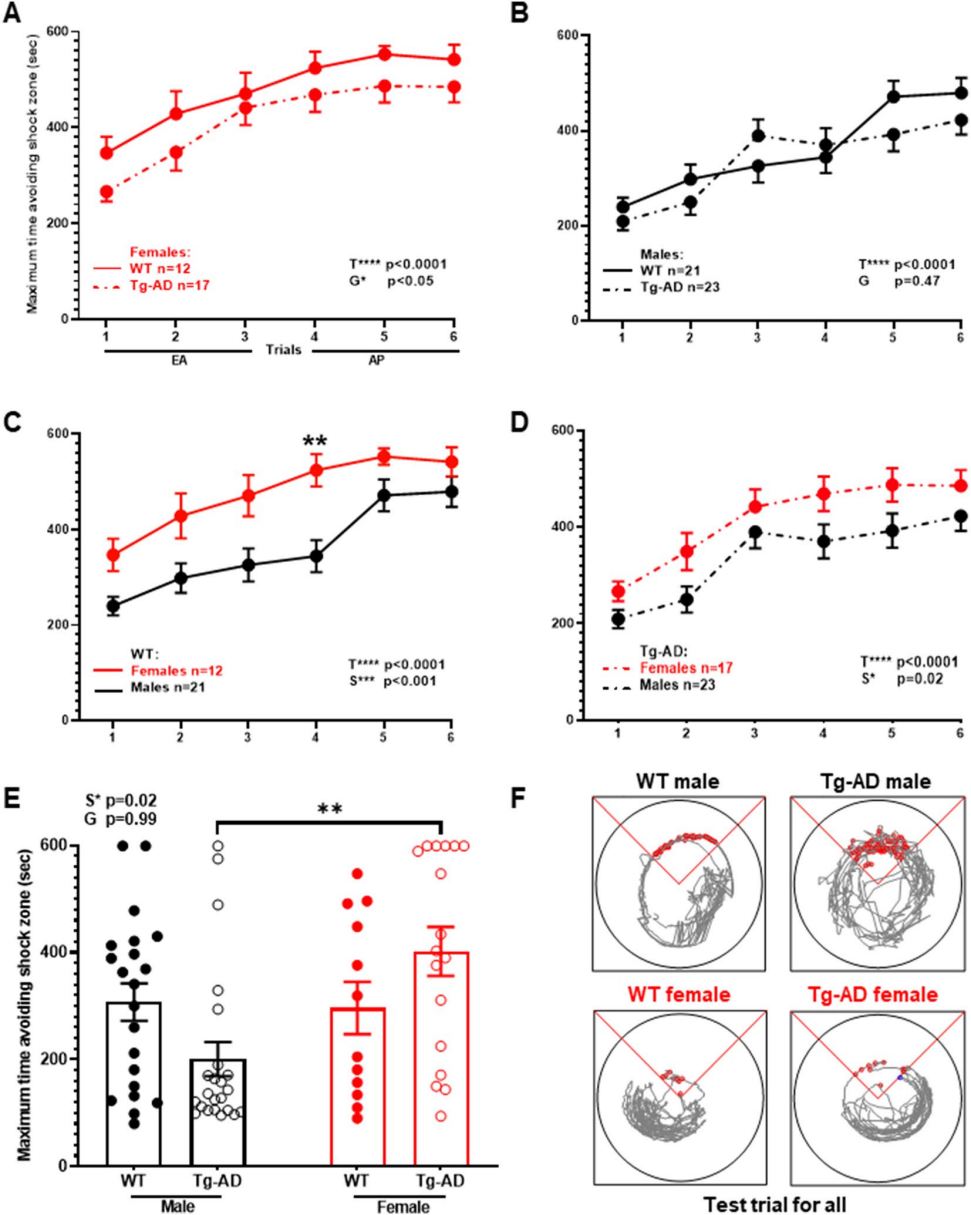
Females outperform males on spatial learning and memory assessed with the active place avoidance task (aPAT). Maximum time to avoid shock zone during training (**A**–**D**). There was an overall difference between WT and TG females (**A**) but not between WT and TG males (**B**). There was no difference in early acquisition (EA) and asymptotic performance (AP) between WT and TG in both sexes. A significant sex effect was observed as WT females performed better than WT males in EA (**C**), and TG females outperform TG males (**D**). The test trial 24 h after training shows overall sex effect as TG females outperform TG males in maximum time to avoid shock zone (**E**). Track tracing of individual rat performances for the test trial across all groups (**F**). Repeated measures two-way ANOVA with Tukey’s post-hoc tests were used in (**A**) through (**D**). Ordinary two-way ANOVA with Tukey’s post-hoc analysis was used in (**E**). Females WT (n = 12), Tg-AD (n = 17); males WT (n = 21), Tg-AD (n = 23). **P < 0.01. *EA* early acquisition, *AP* asymptotic performance. Overall statistical effects across trials (T*), sex (*S), or genotype (G*) are denoted.

**Figure 2:**
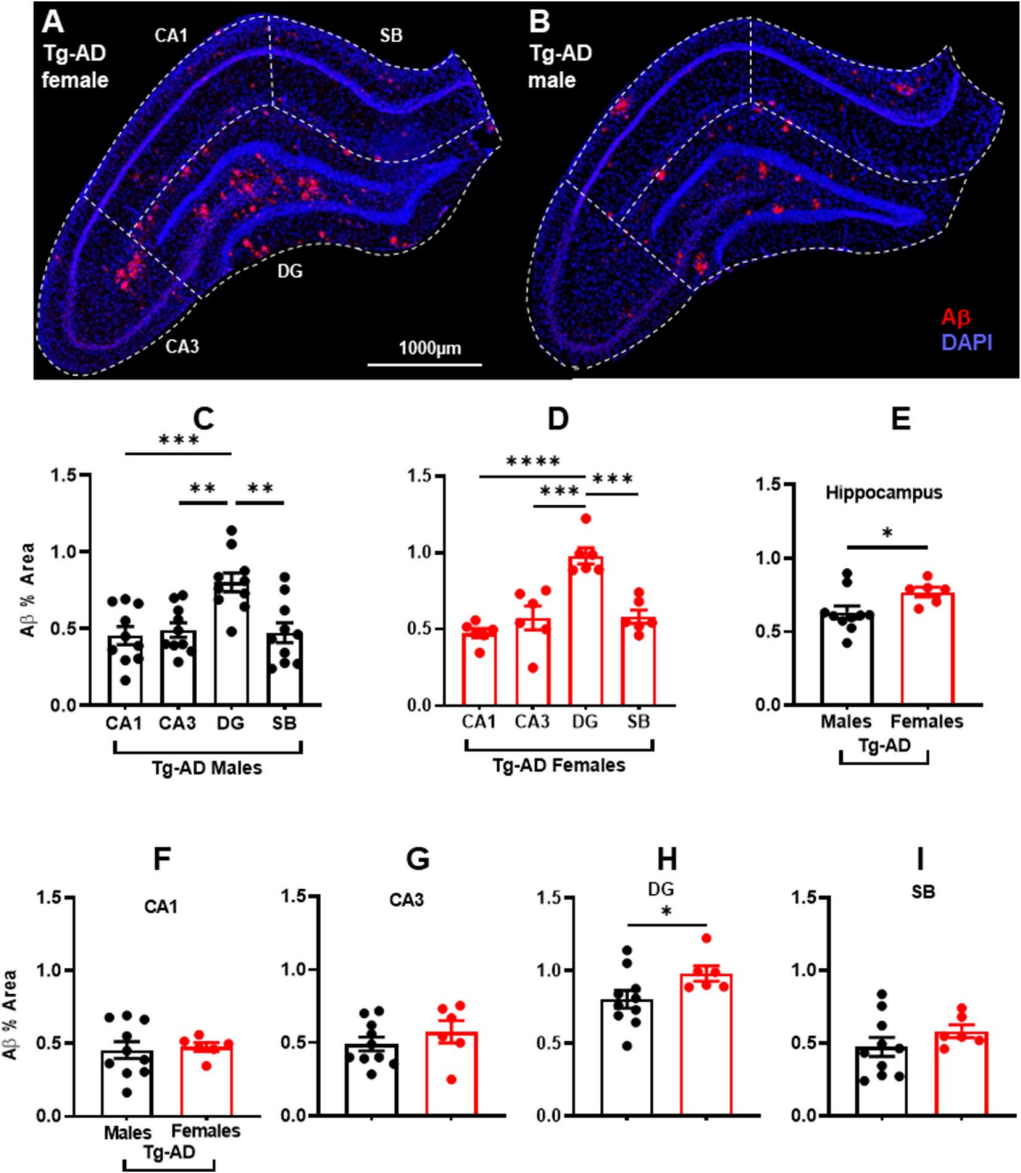
Aβ plaque burden is significantly higher in transgenic females than in males in total hippocampus and dentate gyrus (DG). Representative Aβ (red) and DAPI (blue) staining images for a transgenic female (**A**) and a transgenic male rat (**B**). Scale bar, 1000 µm. Hippocampal region perimeters are depicted in each image by white dashed lines. Plaque burden in transgenic males (**C**) and females (**D**) was significantly higher (~ 1.7-fold) in DG than in other hippocampal regions. Significant increased plaque burden (*P < 0.05) was observed in TG females in whole hippocampus (**E**) and DG (**H**). No difference in plaque burden was observed in CA1, CA3, and SB (**F**,**G**,**I**). Ordinary one-way ANOVA with Tukey’s post-hoc analysis was used in Fig. 1C,D. Unpaired two-tail t-tests with Welch’s corrections were used in 1E through 1I. *P < 0.05. Tg-AD males (n = 10), Tg-AD females (n = 6). *CA* Cornu Ammonis, *DG* Dentate Gyrus, *SB* subiculum.

**Figure 3:**
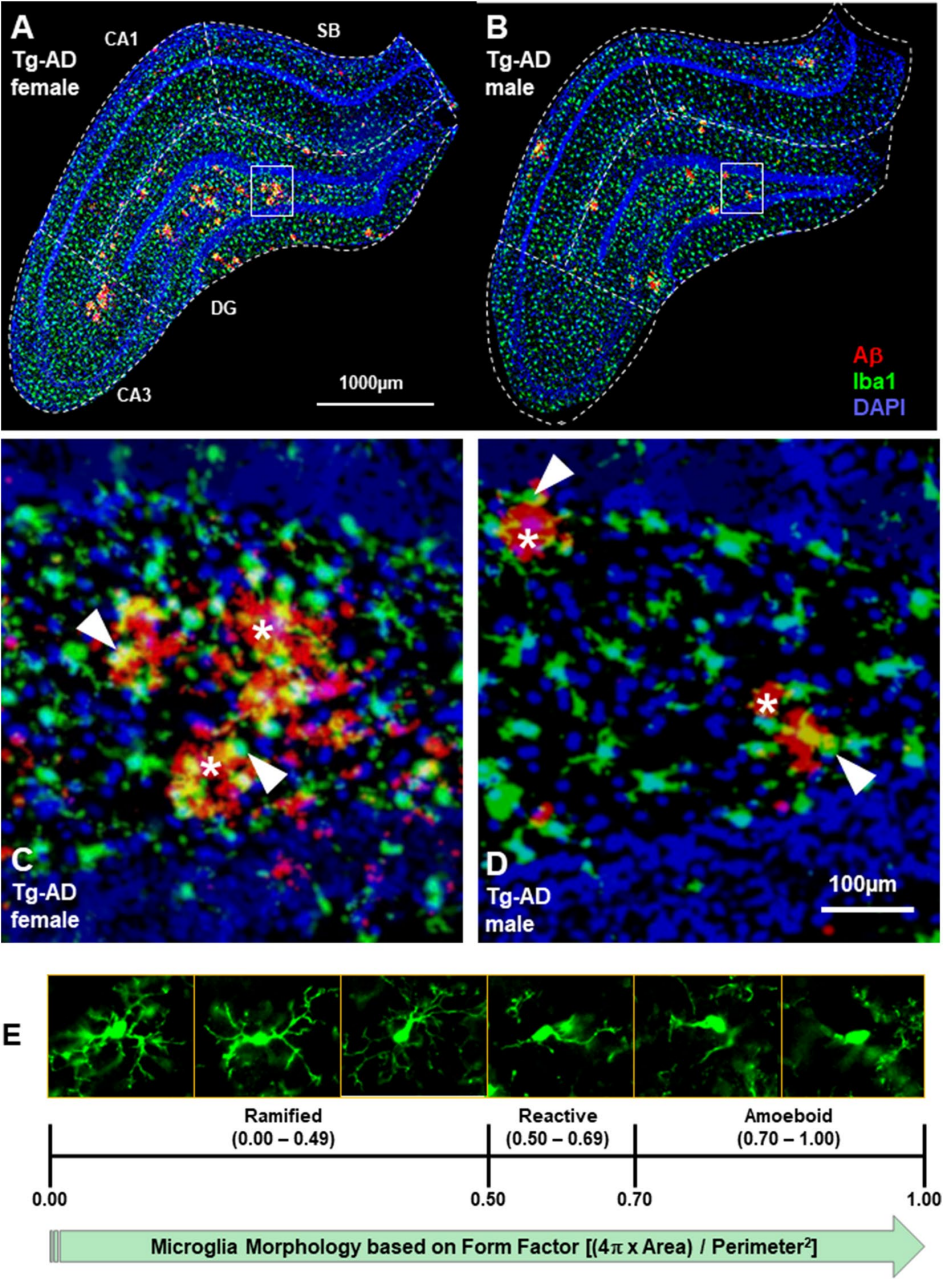

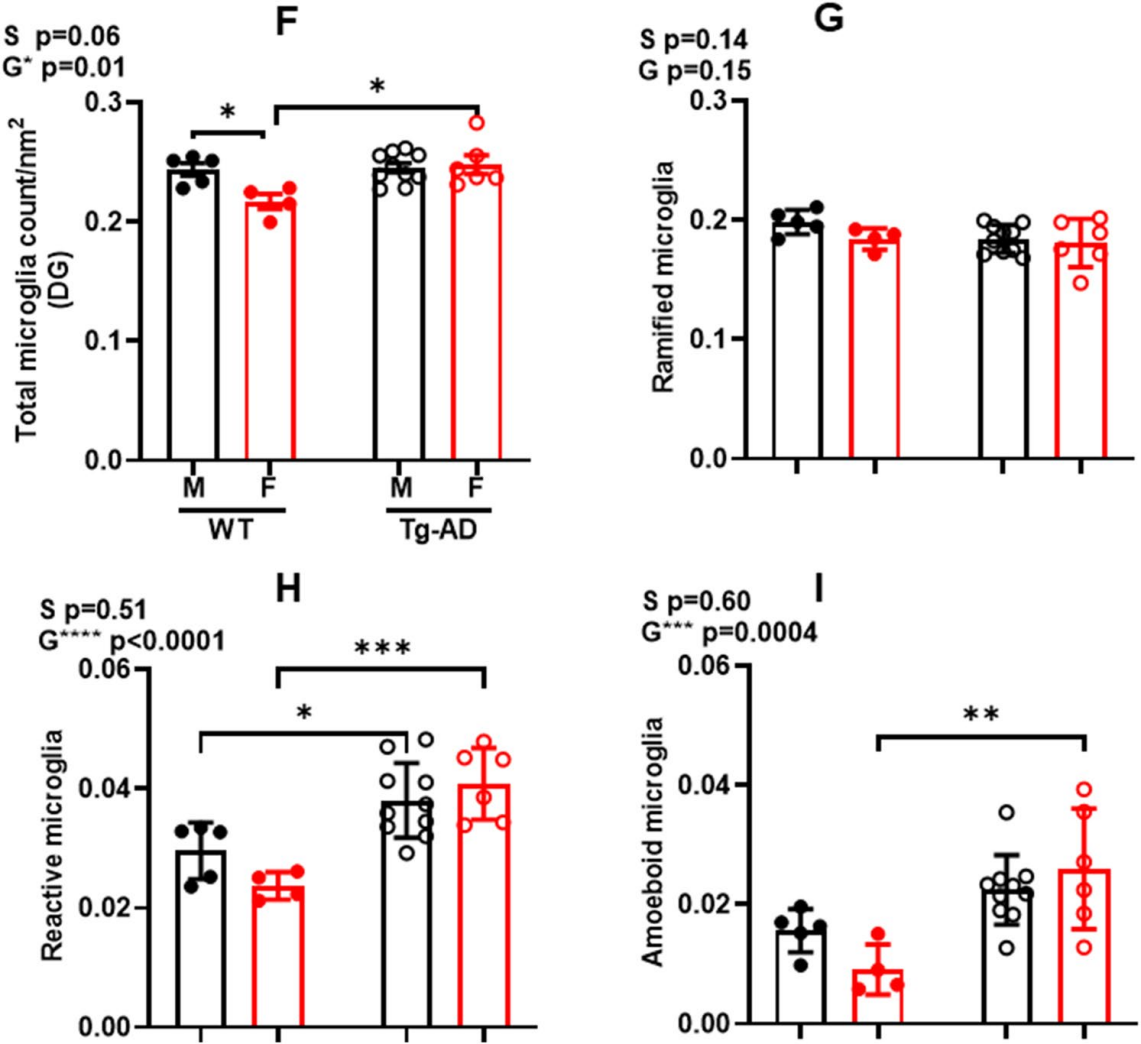
WT female rats have the lowest levels of microglia in the hippocampus compared with the three other groups, Tg-AD females as well as Tg-AD and WT males. IHC analysis for Aβ (red), microglia (green), and nuclei (DAPI, blue) of the right dorsal hippocampus of Tg-AD females (**A**) and Tg-AD males (**B**), 10× magnification, 1000 µm scale bar. Hippocampal region perimeters are depicted in each image by white dashed lines. Bottom panels (**C** and **D**) represent the magnification of the respective small white boxes depicted in (**A**) and (**B**), 100 μm scale bar. White asterisks show the Aβ plaques (red), surrounded by some microglia [white arrows show co-localization (yellow) of microglia and Aβ]. (**E**), The three microglia phenotypes (ramified, reactive and amoeboid, all Iba1+) are based on circularity (form factor) as explained under material and methods. WT females have significantly fewer microglia (counts/nm^2^) in the whole hippocampus (right dorsal) than the other three groups of rats (**F**). The number of ramified microglia was not significantly different in the four groups of rats (**G**). The number of reactive (**H**) microglia was significant lower in WT females and males compared to Tg-AD females and males. The number of amoeboid (**I**) microglia for WT females was significantly lower compared to Tg-AD females and males. Ordinary two-way ANOVA with Tukey’s post-hoc tests were used in (**F**) through (**I**). *P < 0.05, **P < 0.01, ***P < 0.001. Females WT (n = 4), Tg-AD (n = 6); males WT (n = 5), Tg-AD (n = 10). *CA* Cornu Ammonis, *DG* Dentate Gyrus, *SB* subiculum.

**Figure 4:**
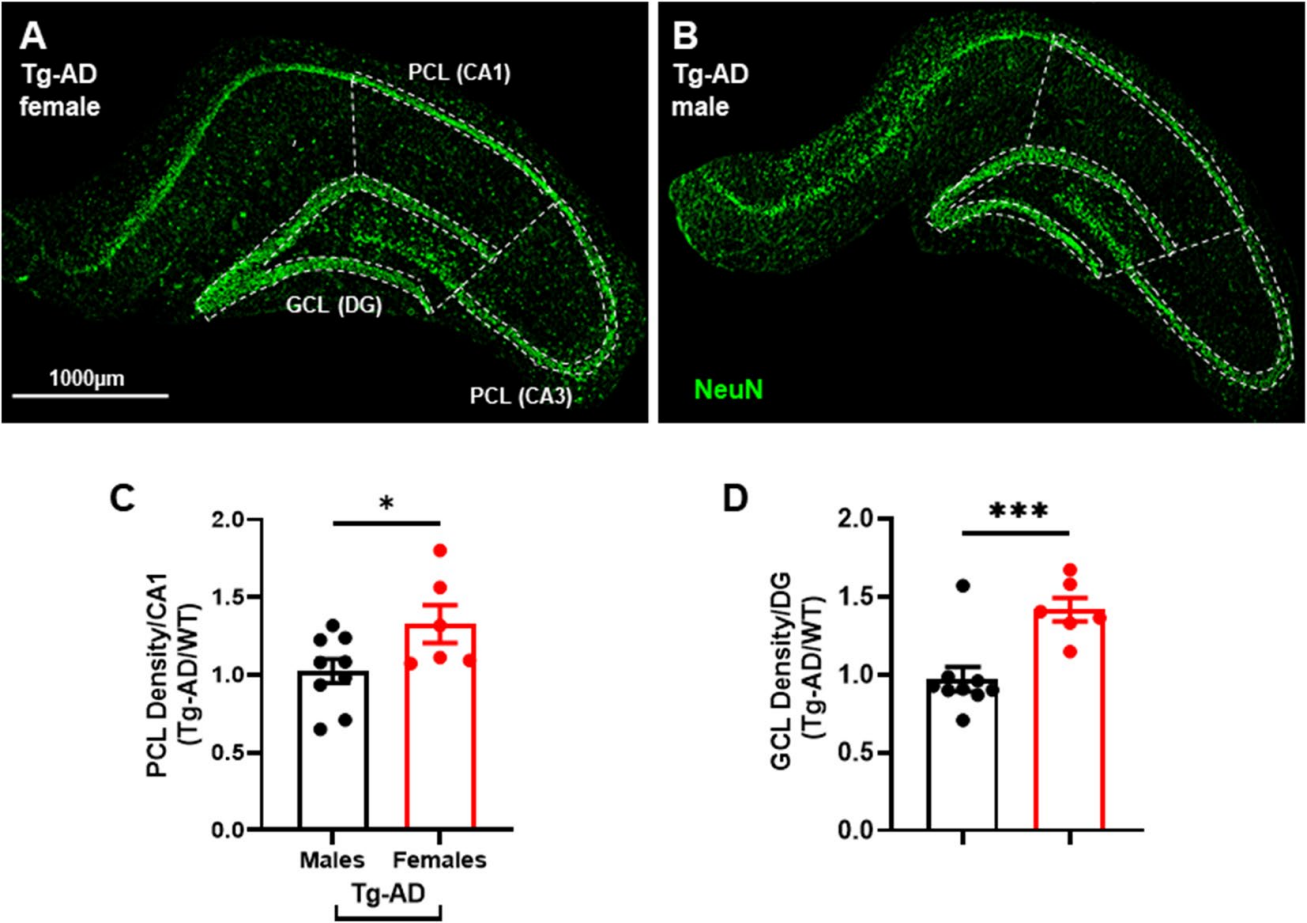
Tg-AD females have a higher hippocampal neuronal density than Tg-AD males. IHC analysis for mature neurons (green) of the right dorsal hippocampus of Tg-AD females (**A**) and Tg-AD males (**B**), 10× magnification, 1000 μm scale bar. Hippocampal pyramidal (PCL) and granule (GCL) neuronal cell layers are highlighted with white dashed lines. Tg-AD female rats had significantly higher neuronal density (NeuN staining) in the (**C**) PCL (CA1) and (**D**) GCL (DG) hippocampal regions compared to Tg-AD males. Unpaired one-tail t-tests with Welch’s corrections were used for comparison. *P < 0.05, ***P < 0.001. Tg-AD males (n = 9), Tg-AD females (n = 6). *CA* Cornu Ammonis, *DG* Dentate Gyrus.

**Figure 5:**
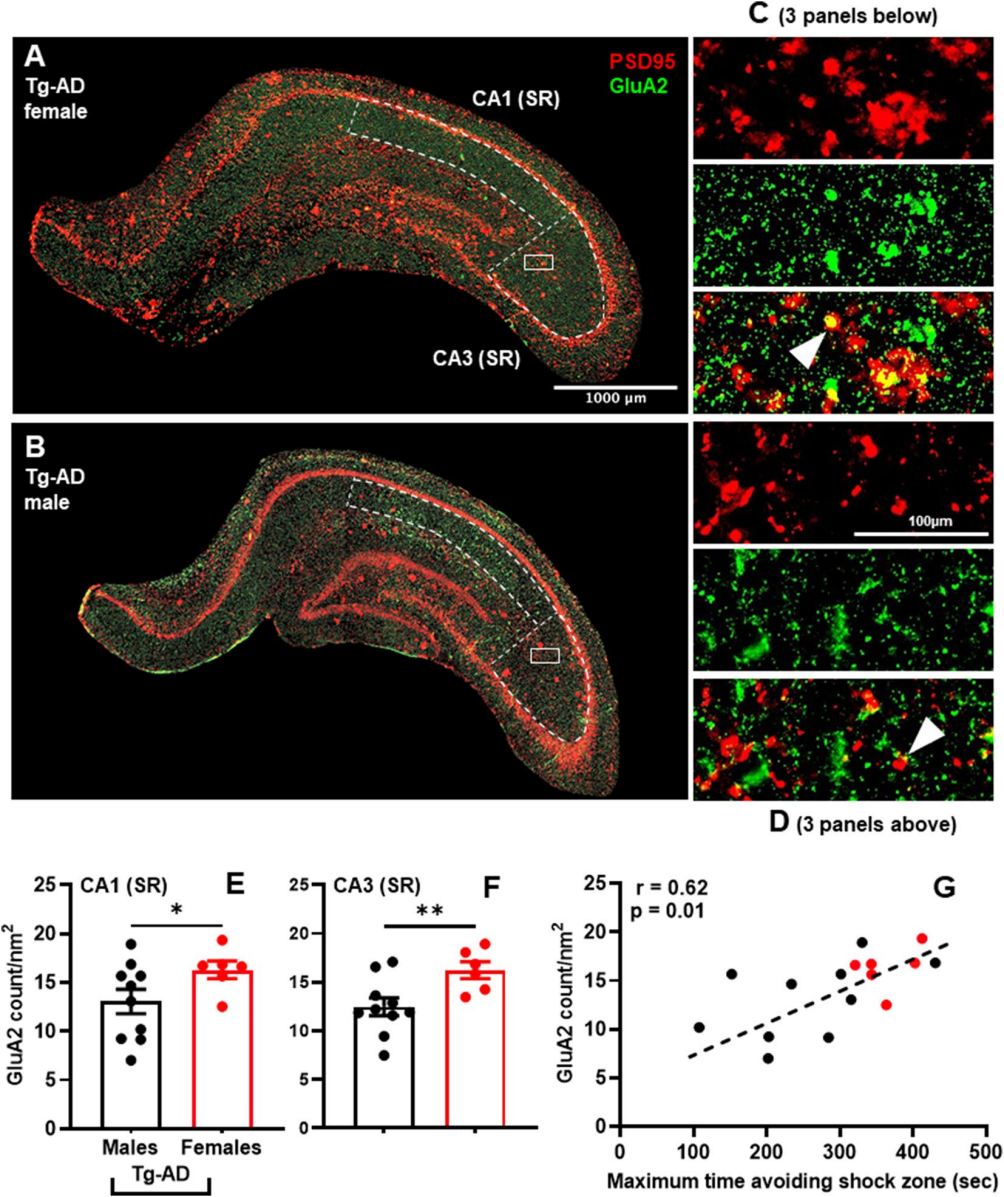
Tg-AD females exhibit increased levels of GluA2 in the hippocampus compared to Tg-AD males. IHC analysis for the AMPA receptor GluA2 subunit (green) and the scaffolding protein PSD95 (red) of the right dorsal hippocampus of Tg-AD females (**A**) and Tg-AD males (**B**), 10× magnification, 1000 µm scale bar. Smaller panels on the right (**C** and **D**) represent the magnification of the respective small white boxes depicted in (**A**) and (**B**), 100 µm scale bar, showing the two stains (GluA2, green and PSD95, red) separately and co-localized (yellow, white arrows). Hippocampal CA1 SR and CA3 SR subregions are highlighted with white dashed lines. Tg-AD female rats had significantly higher GluA2 levels in the (**E**) CA1 (SR) and (**F**) CA3 (SR) hippocampal subregions compared to Tg-AD males. Unpaired one-tail t-tests with Welch’s corrections were used for the comparison. *P < 0.05, **P < 0.01. (**G**) GluA2 levels (y-axis) directly correlates with cognitive behavior (x-axis). The correlation was evaluated by linear regression calculating the Pearson correlation coefficient. Tg-AD males (n = 9), Tg-AD females (n = 6). *CA* Cornu Ammonis, *SR* stratum radiatum.

**Figure 6:**
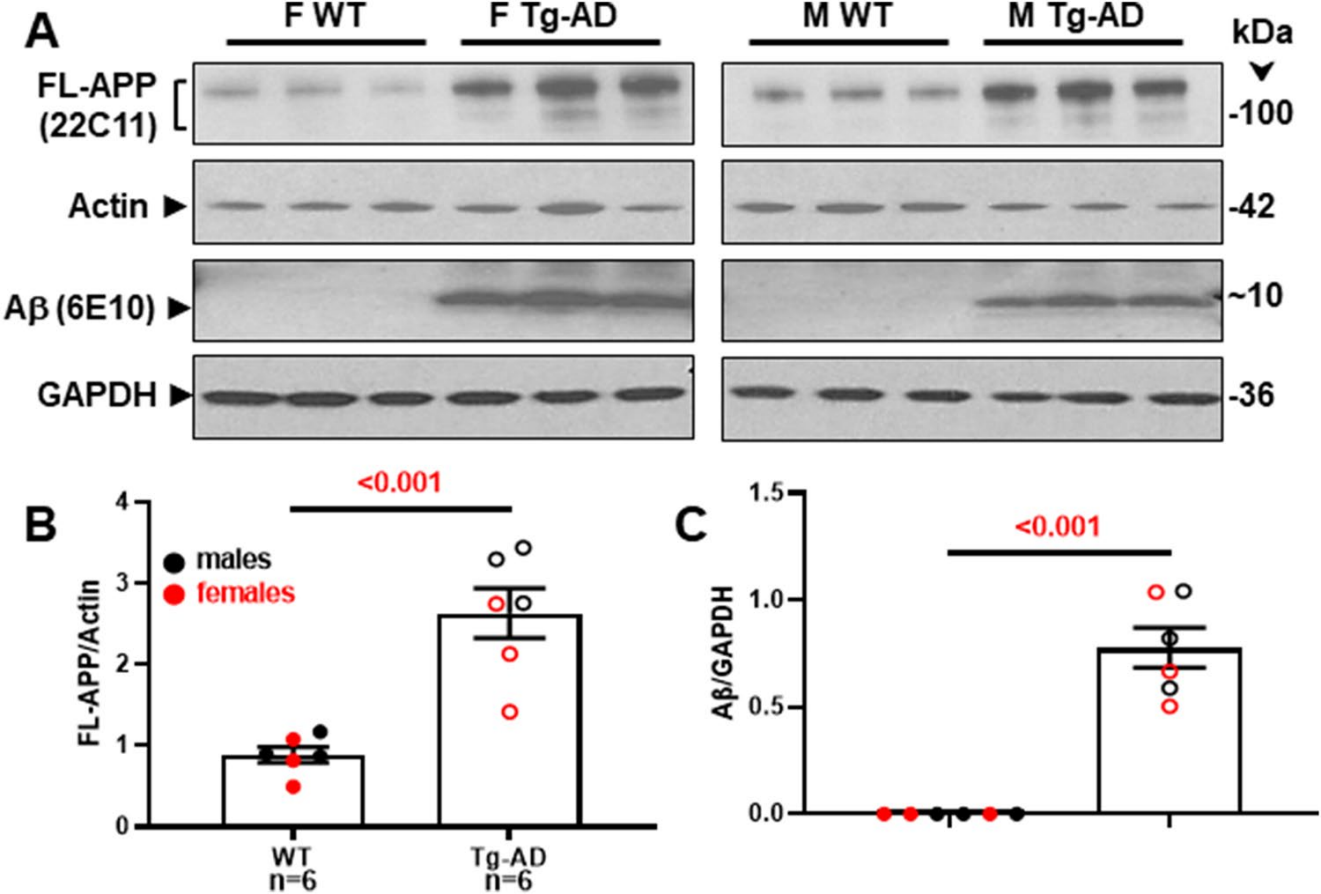
Tg-AD rats show enhanced FL-APP and Aβ peptide levels in the hippocampus at 9-months of age. (**A**) FL-APP (top panels) and Aβ levels (third panels from the top) were assessed by western blot analysis in whole left hippocampal (combined ventral and dorsal) homogenates from 9-month WT and Tg-AD female (F, 3 of each genotype) and male (M, 3 of each genotype) rats. Actin (second panels from the top) and GAPDH (bottom panels) detection served as the respective loading controls. FL-APP (**B**) and Aβ (**C**) levels were semi-quantified by densitometry. Data represent the percentage of the pixel ratio for FL-APP and Aβ over the respective loading controls for Tg-AD compared to WT (value of one). Values are means ± SEM from 6 rats per genotype (males and females combined). Significance (p values shown on graphs) was estimated by an unpaired one-tail t-tests with Welch’s corrections. There was no significant difference between males and females for both genotypes. The tissue samples used to detect each protein and their respective control were derived from the same experiment and analyzed on the same gel/blot. Blots were processed in parallel. The original/uncropped blots for each detection are provided in the Supplementary information file with each blot labeled according to the protein detected.

All data are represented as the mean ± SEM. Statistical analyses were performed with GraphPad Prism 9 (GraphPad Software, San Diego, CA). All *P* values, SEMs and *t*-statistics are shown on graphs. Welch’s unpaired one-tailed *t* test was used to compare means between the two groups (Tg-AD males and Tg-AD females) for IHC (Figs. 2, 4, 5) and WB (Fig. 6). Ordinary one-way ANOVA with Tukey’s post-hoc analysis was used to compare plaque burden in the four hippocampal regions (Fig. 2C,D). A two-way repeated measure analysis of variance (ANOVA) with multi-factor comparisons for aPAT (Fig. 1) and microglia (Fig. 3) were performed followed by Tukey’s post hoc tests. For image quantification, normalization of pixel intensity values across images was done utilizing the rolling ball algorithm^36^.

### Ethics approval

All animal experiments were performed in compliance with the regulations of the Institutional Animal Care and Use Committee at Hunter College.

## Results

### Female Tg-AD and WT rats outperform male counterparts at 9-months of age in spatial learning and memory

A two-way repeated ANOVA between WT and Tg-AD females (Fig. 1A) show an overall effect of training [F_(4.2, 114)_ = 14.67, P < 0.0001] and genotype [*F*_(1,24)_ = 13.83, *P* < *0.05*]. The post-hoc analyses were not significant. We next analyzed the learning curve across two components: early acquisition (trials 1–3) and asymptotic performance (trials 4–6). Analyses for effects during early acquisition (EA) and asymptotic performance (AP) were not significant (EA, F_(1,42)_ = 0.032, P = 0.87; AP, F(_1,42_) = 0.96, P = 0.33). No differences were detected between WT and TG males across all 6 trials (overall) or in early acquisition and asymptotic performance [Fig. 1B, F_(1,24)_ = 0.53, *P* = 0.47; EA, F_(1,42)_ = 0.03, P = 0.87; AP, F_(1,42)_ = 0.96, P = 0.33)]. Analysis of WT males vs females (Fig. 1C) show an overall effect of training (F _(4, 130)_ = 14.88, P =  < 0.01); and sex (F _(1,31)_ = 17.87, P < 0.001; with post-hoc differences at trial 4 (t = 179, p = 0.005). Likewise, analysis of TG males vs females (Fig. 1D) shows an overall effect of training (F_(4.6, 175)_ = 22.73, P < 0.0001); and sex (F _(1,38)_ = 6.08, P = 0.02) with no post-hoc differences. Figure 1F shows the track tracing of individual rat performances for the test trial across all groups. These results together indicate that female WT and TG animals outperformed their genetic matched males.

### Tg-AD females exhibit greater Aβ plaque burden than Tg-AD males in whole hippocampus and DG

Tg-AD rats express human APP and accumulate Aβ peptides forming extracellular plaques^25^. An immunohistochemical analysis for Aβ (antibody 4G8, 1:1000, BioLegend, cat# 800708, amino acid residues 17–24) in WT and Tg-AD rats showed no plaques in WT rats of both sexes (not shown). Significant plaque load was observed in both male and female Tg-AD rats showing that amyloid pathology is present at 9-months of age (Fig. 2A,B, amyloid plaques, red; DAPI, blue).

Since AD pathology spreads from the entorhinal cortex (layer II) → dentate gyrus → CA3 → CA1^3^, we compared plaque load in four hippocampal regions, CA1, CA3, DG, and SB. All hippocampal regions presented amyloid plaques, but in different amounts. One-way ANOVA followed by Tukey’s post hoc analysis showed a regional effect, in that amyloid plaque burden was at least 1.6-fold higher in the DG region than in any of the other hippocampal regions both in male (Fig. 2C) and female Tg-AD rats (Fig. 2D). The significance of the regional plaque loads in the CA1, CA3 and SB of males and females is shown relative to DG in Supplemental Table 1. These data show that the spread of pathology regarding amyloid plaques recapitulates early-stage AD.

We also compared the sex differences in amyloid plaque pathology, since sex discrepancies were reported for male and female patients in AD development and in other rodent models of AD^37,38^. Our results show a significant increase in the % area amyloid plaques in female Tg-AD rats compared to male littermates in the whole hippocampus (Fig. 2E) and in the hippocampal DG region (Fig. 2H). In other hippocampal regions no sex-dependent differences were observed in amyloid plaque load (Fig. 2F,G,I).

### Tg-AD females show increased microgliosis in whole hippocampus and DG compared to WT females, complimentary to Aβ plaque burden

We performed immunohistochemical staining for Iba1 and the analysis showed microgliosis across CA1, CA3, DG and SB in WT and Tg-AD male and female rats (Fig. 3A,B, whole hippocampus, C and D respective high magnification panels, shown for Tg-AD rats; amyloid plaques, red; microglia, green; amyloid plaques/microglia co-localization, yellow, indicated by white arrow heads). Two-way ANOVA of micoglia counts/nm2 in the DG (Fig. 3F) shows an overall effect of genotype (F_(1, 21)_ = 7.209, P = 0.0139) with significant post-hoc differences between female TG vs WT (t = 4.750, P = 0.0146) and between WT males vs females (t = 3.95, P = 0.0498).

Microglia can be divided into three forms according to their functions and cell body circularity: ramified, reactive and amoeboid^34^ (Fig. 3E). Highly ramified microglia can change to an amoeboid form on pathological stimulation^39^. Ramified microglia, which are considered the “homeostatic” neuroprotective form^40^, were most abundant followed by reactive and then amoeboid. There no significant effects from a two-way ANOVA for ramified microglia counts/nm^2^ (Fig. 3G). Reactive microglia (Fig. 3H) represent the activated pro-inflammatory form of microglia^40^. Two-way ANOVA shows an overall effect on genotype (F _(1, 21)_ = 29.90, P < 0.0001) with post-hoc differences between female WT vs TG (t = 6.784, p = 0.0005); and male WT vs TG (t = 3.957, p = 0.0489). Analysis of amoeboid microglia (Fig. 3I) which are considered to be the neurotoxic and overactive form^40^, shows an overall effect on genotype (F _(1, 21)_ = 17.81, p = 0.0004) with post-hoc differences between females TG vs WT (t = 5.568, p = 0.0039). These findings are consistent with the increased microgliosis observed in AD patients where active microglia extend their processes into the plaque core and cluster around amyloid plaques as shown in Fig. 3C,D^41–43^.

### Tg-AD males display increased neuronal loss in CA1 and DG compared to Tg-AD females

We assessed neuronal density across hippocampal regions CA1 and CA3 pyramidal cell layers (PCL), as well as DG granule cell layer (GCL) with NeuN (green) to quantify mature neurons (Fig. 4A,B, shown for Tg-AD rats of both sexes). We detected a significant difference in neuronal density between Tg-AD males vs females only in the CA1 pyramidal cell layer (22.7% less, *t* = 2.09, *p* = 0.033) and DG granule cell layer (31.5% less, *t* = 4.05, p = 0.0007) (Fig. 4C,D). Data were normalized to their WT controls for simplicity. There were no differences in WT males vs females for CA1 PCL, CA3 PCL or DG GCL [CA1 PCL (t = 0.0826, p = 0.4688); CA3 PCL (t = 0.1832, p = 0.4308); DG GCL (t = 2.0, p = 0.5)]. These finding suggest that mechanisms responsible for neuronal loss are independent of plaque formation and microglia levels.

### Increased levels of GluA2 in the hippocampus of Tg-AD females complement their superior cognitive performance compared to Tg-AD males

The levels of the AMPA receptor GluA2 subunit at synapses are important for spatial memory^44,45^, as is the AMPA receptor trafficking regulated by the scaffolding protein PSD95 at glutamatergic synapses^46^. We performed IHC analyses of the hippocampus for GluA2 and PSD95 (Fig. 5A,B, whole hippocampus, C and D respective high magnifications panels, shown for Tg-AD rats; PSD95, red; GluA2, green; PSD95/GluA2 co-localization, yellow, indicated by white arrow heads). The levels of GluA2 are higher in Tg-AD females than in Tg-AD males, specifically at the CA1 SR subregion (Fig. 5E, t = 2.10, *p* = 0.037) and CA3 SR subregion (Fig. 5F, t = 2.99, *p* = 0.005) depicted by the dashed lines in Fig. 5A,B. There were no differences in GluA2 detected in any subregion for WT males vs females. Results show that higher levels of GluA2 in Tg-AD females correlate with improved spatial learning, as shown in Fig. 5G. These findings suggest that higher regional hippocampal GluA2 levels in Tg-AD females contributes to spatial memory maintenance independently of their higher plaque load.

### Tg-AD rats show enhanced FL-APP and Aβ peptide levels in the hippocampus

Tg-AD rats express Full length APP (FL-APP) at 9-months of age at a significant higher level than their WT counterparts (Fig. 6A, top panel). This trend was observed in males (n = 3 for each genotype) and females (n = 3 for each genotype), and the values were normalized for actin (Fig. 6B, p < 0.001). FL-APP was detected with the mouse monoclonal antibody 22C11, which reacts with human and rat, as well as other species (manufacturer’s specifications).

Aβ levels were assessed in the same samples of rat hippocampal tissue with the mouse monoclonal antibody 6E10, which has a threefold higher affinity for human APP and Aβ compared to the rat counterparts (manufacturer’s specifications). Aβ peptides were detected in male and female Tg-AD rats but not in the WT littermates, as shown in Fig. 6A [third panels labeled with Aβ (6E10)], with values normalized for GAPDH in Fig. 6C (p < 0.001). The presence of Aβ plaques in all 9-month Tg-AD rats was confirmed by IHC analysis with the mouse monoclonal antibody 4G8 (1:1000, BioLegend, cat# 800708, amino acid residues 17–24), as shown for one female and one male Tg-AD rat in Fig. 2A. This antibody has a greater affinity for human Aβ.

These data verify that all the findings in Figs. 1, 2, 3, 4, 5 were with males and females that exhibit equal levels of APP and Aβ [Abeta (t = 0.3972, p = 0.36) and APP (F _(2, 6)_ = 0.4373, P = 0.66)]. Thus, the differences that we see the between sexes cannot be attributed to differences in APP or Aβ expression levels.

## Discussion

AD affects both working and long-term memory, as loss of memory is among the first symptoms reported by AD patients^47^. This cognitive deficit is expressed by the Tg-AD rat model as a significant spatial memory impairment relative to WT animals^25^. We assessed cognitive ability of the Tg-AD rats via aPAT, to identify genotype- and sex-dependent spatial memory impairment. The higher the value, the longer it took the rat to enter the shock zone for the first time, supporting a sound level of spatial memory and recognition. Improved performance in the aPAT assessment is indicative of sound memory, as hippocampal inactivation impairs performance in this task^48^. We found significant deficits only in Tg-AD females compared to wild type females, but not between the two male genotypes suggesting that 9-months of age may be too early to observe significant cognitive decline via aPAT using male Tg-AD rats.

Previous findings in Tg-AD rats reported neurocognitive impairments in 5-month rats (pre pathology) by means of delayed non-match-to-sample task^49^. Although we observed poorer performance in Tg-AD compared to WT littermates, the difference was not significant. This result suggests that 9-months of age may be too early to observe significant cognitive decline via aPAT using this Tg-AD rat model. Neurocognitive impairment for this rat model was detected at 15 months of age using a Barnes maze^25^ and at 10–11 months of age using the Morris water maze^50^. However, these two mazes are stressful on their own to rats causing an increase in plasma corticosterone. Thus, these mazes may cause test-induced responses that, by themselves, can affect cognition^51^ and add an additional stress factor to the AD pathology.

In a previous study, Tg-AD rats showed spatial navigation impairment at 4 to 5 months of age using Active Allothetic Place Avoidance (AAPA), which increased at 6 to 7 months^52^ without a sex effect. In our studies, female WT and Tg-AD rats scored better with the aPAT assessment than their male littermates. Females of both genotypes exhibited superior latency to 1st entrance, latency to 2nd entrance, and maximum time to avoid shock than males. These aPAT results are in contrast with the immunohistochemical analysis discussed below, showing higher amyloid plaque load and gliosis in transgenic female rats. This suggests that the age-dependent progression of cognitive impairment is slower in females than in males.

We show a sex-dependent amyloid plaque load in 9-month Tg-AD rats with a significantly higher % area level of plaques in females. The accumulation of Aβ plaques is thought to be one of the main events of neurodegeneration in AD that could contribute to synaptic dysfunction^53^, neurofibrillary tangles, and neuronal loss causing impaired memory and cognitive dysfunction. Indeed, 9-month-old Tg-AD rats have plaques in the entorhinal cortex, hippocampus and cortical arterioles^54^ as well as the DG and CA1 regions^55^ along with a doubling of tau phosphorylation at Ser202/Thr205 and a 1.5-fold increase in tau phosphorylation at Thr231^54,56^. At 6 months of age, TgF344-AD rats have reduced basal synaptic transmission, increased Aβ oligomers, hyperphosphorylated tau and activated microglia and astrocytes^25,55^ as well as reduced tyrosine hydroxylase positive axons in the hippocampus^29,57^. Since neurofibrillary tangles in the Tg-AD rats are only detected around 15-months of age^25^, they were not included in our studies.

In other AD animal models, females display more amyloid plaques at different ages^58^ and higher levels of tau phosphorylation at late stages^38^. However, Aβ levels in female APP/PS1 mice were significantly higher, but unlike our findings, corresponded to reduced memory test performance^59^. It is possible that Aβ deposits might have a greater effect on cognition in these AD models.

The sex differences exhibited by the Tg-AD rats is consistent with that observed in AD patients and rodent models^37,38^. The neuronal loss in male and female rats in the CA1 and DG regions is consistent with findings that AD patients have faster progression of hippocampal atrophy. In our studies, males show greater neuronal loss than females. Nevertheless, AD and other dementias disproportionately affect women^60^ due to a faster progression of hippocampal atrophy in females with AD^59^ and regional hippocampal differences in neuronal loss^37,38^.

Additional sex differences in the Tg-AD rat model included reports that basal synaptic transmission deficits at CA3-CA1 synapses occur at 9 months in males and 12-months in females^55^, supporting a neuroprotective role for estrogen in this rat model. However, other studies showing spatial memory impairment in Tg-AD rats at 6- to 7-months were without sex differences^52^.

As AD involves memory loss, we examined various hippocampal regions in the 9-month Tg-AD rats for amyloid pathology and found amyloid plaques in the CA1, CA3, DG and SB regions. There was a statistically significant increase in plaque number in Tg-AD females only in the DG hippocampal region. While amyloid pathology was prominent only in the DG region at 9-months of age, additional studies are needed to track plaque progression throughout the hippocampus as the transgenic rats age. It is likely that the limited AD pathology detected in the hippocampus of these 9-months transgenic rats is not sufficient to impair cognition. The behavioral deficits develop at a later age, when pathology is more advanced.

AD is also associated with chronic neuroinflammation caused by overactive microglia^17^. Since gliosis is implicated in the pathology of AD, we hypothesized that microglia morphology may be altered. The state of microgliosis in WT and Tg-AD rats at 9-months of age displayed increased microglia count in the hippocampus with a significant genotype effect on microglia numbers and % area between WT and Tg-AD females. Microglia are recruited to amyloid plaques for phagocytosis^21^ and exhibit a variety of morphologies that can be associated with their particular functions^34^. An examination of individual microglia silhouettes revealed that Tg-AD rats of both sexes had more reactive microglia than WT but no sex differences. These findings are consistent with the increased levels of amyloid plaques in Tg-AD rats and findings that AD patients show increased gliosis. Interestingly, the levels of amoeboid microglia which are considered to be neurotoxic, was significantly greater in Tg-AD female than WT females.

Microglia are recruited to the site following a threat to the CNS, such as amyloid plaques, and extend their processes into the plaque core perform phagocytosis to clear plaques^21^. The extensive gliosis detected in the Tg-AD females may further exacerbate neuroinflammation, leading to neuronal loss and increased AD pathology in females^22–24^. Thus, higher levels of neuroinflammation may contribute to the sensitively and disproportionate effect of AD on women, and be manifested as the higher plaques and microglia. In contrast, female Tg-AD rats had more neurons than Tg-AD males which may explain the greater cognitive performance observed in females.

In this study, we established that 9-month transgenic female rats exhibited higher levels of amyloid plaques but performed better than males in a hippocampal-dependent cognitive task. Estrogen is known for being neuroprotective but plays an unknown function in AD^61^. Estrogen was shown to improve spatial learning in APP/PS1 mice without affecting Aβ plaque accumulation^62^, and protect against apoptosis induced by Aβ in hippocampal neurons^63^. In gonadectomized rats estrogen treatments resulted in a twofold higher increase in hypothalamic GluA2/3 expression in females^64^, and was shown to enhance learning and memory by stimulating GluA2 trafficking to mushroom spines^33^.

Our studies show that the improved cognition in Tg-AD females was paralleled by increased levels of GluA2. Studies showed that a significant correlation exists between retention tests scores and synaptic GluA2 levels in the hippocampus^65^. Similarly, an improvement in spatial memory in the hippocampus in Tg 2576 mice was associated with increased expression of both GluA1 and GluA2 at synapses without affecting Aβ protein levels^66^. Mitigation of memory deficits resulted in increased GluA1 and GluA2 levels in the 3xTg-AD mouse model, suggesting that AMPA receptors play a critical role in synaptic plasticity and memory^67^. Nevertheless, GluA2 is significantly expressed in the human post-mortem hippocampus of AD patients relative to controls, specifically in the stratum moleculare of the DG^68^.

In conclusion, our results suggest that female Tg-AD rats possess a protective mechanism against cognitive dysfunction but not other pathological deficits in early-stage AD. This mechanism involves an increase in GluA2, suggesting that AMPA receptor activity preserves synaptic plasticity in early AD. This hypothesis needs to be confirmed in future studies.

## Supplementary Information

Supplementary Information.

## Data Availability

All data generated for this study are provided in the manuscript. Data supporting the findings of this manuscript are available from the corresponding authors upon request.

## Abbreviations

Aβ: Amyloid-beta
AD: Alzheimer’s disease
APP: Amyloid precursor protein
BACE1: β-Secretase 1
CA: Cornu ammonis
DG: Dentate gyrus
GAPDH: Glyceraldehyde 3-phosphate dehydrogenase
GCL: Granule cell layer
HL: Hilar
Iba1: Ionized calcium binding adaptor molecule 1
NeuN: Neuronal nuclei, neuronal marker
PCL: Pyramidal cell layer
SB: Subiculum
SEM: Standard error of the mean
SR: Stratum radiatum
TgF344-AD = Tg-AD: Transgenic rat model of Alzheimer’s disease
WT: Wild type
