# Supplementary material for: Females exhibit higher GluA2 levels and outperform males in active place avoidance despite increased amyloid plaques in TgF344-Alzheimer’s rats
Source: Sci Rep. 2022 Nov 9;12:19129. doi: 10.1038/s41598-022-23801-w (PMC9646806; doi:10.1038/s41598-022-23801-w)
Supplement: Supplementary file 1 — Supplementary Information. [file 41598_2022_23801_MOESM1_ESM.pdf]

Supplemental Table 1 Plaque load across hippocampal regions

	F Tg-AD mean ± SEM n = 6	*p*-value (DG vs other)	M Tg-AD mean ± SEM n = 10	*p*-value (DG vs other)
**SB**	0.58 ± 0.0.4	0.0002***	0.47 ± 0.07	0.002**
**CA1**	0.48 ± 0.03	<0.0001****	0.46 ± 0.06	0.0009***
**CA3**	0.57 ± 0.08	0.0002***	0.49 ± 0.05	0.003**
**DG**	0.98 ± 0.05	-	0.80 ± 0.06	-

Values represent the percent of the signal (Aβ+) detected within a specific area (= 100%, μm^2) as explained under materials and methods. Abbreviations: Tg-AD, transgenic rat model of Alzheimer's disease; SEM, standard error of the mean; SB, subiculum; CA, cornu ammonis; DG, dentate gyrus. Statistical analysis: one way ANOVA and Tukey's multiple comparison.

Supplemental Table 2 Antibodies used for IHC and WB analyses of hippocampal tissue

Antibody	Company	Catalog Number	Species/Type	Dilution	Assay
PRIMARIES					
**Aβ (4G8)**	Biolegend	#800708	Mouse Monoclonal	1:1000	IHC
**Aβ (6E10)**	Biolegend	#SIG-39320	Mouse Monoclonal	1:2000	WB
**FL-APP (22C11)**	Millipore Sigma	#MAB348	Mouse Monoclonal	1:2000	WB
**Iba1**	Fujifilm WAKO	#019-19741	Rabbit Polyclonal	1:500	IHC
**NeuN**	Millipore Sigma	#ABN91	Chicken Polyclonal	1:250	IHC
**β-Actin (AC-74)**	Sigma-Aldrich	#A2228	Mouse Monoclonal	1:20000	WB
**GluR2 (6C4)**	Millipore Sigma	#MAB397	Mouse Monoclonal	1:800/1:3000	IHC/WB
**GluR1**	Millipore Sigma	#ABN241	Rabbit Polyclonal	1:2000	WB
**PSD95 (D27E11)**	Cell Signaling	#MAB3450	Rabbit Monoclonal	1:200/1:1000	IHC/WB
SECONDARIES					
**Alexa Fluor 488, Goat anti-Rabbit IgG (H+L)**	ThermoFisher	#A-27034	Rabbit Secondary	1:250	IHC
**Alexa Fluor 488, Goat anti-Mouse IgG (H+L)**	ThermoFisher	#A-11029	Mouse Secondary	1:250	IHC
**Alexa Fluor 568, Goat anti-Mouse IgG (H+L)**	ThermoFisher	#A-11031	Mouse Secondary	1:250	IHC
**Alexa Fluor 568, Goat anti-Rabbit IgG (H+L)**	ThermoFisher	#A-11036	Rabbit Secondary	1:250	IHC
**DyLight 350, Goat anti-Chicken IgY (H+L)**	ThermoFisher	# SA5-10069	Chicken Secondary	1:100	IHC
**Goat Anti-Rabbit IgG (H + L)-HRP**	Cell Signaling	#7074	Rabbit Secondary	1:2000	WB
**Goat Anti-Mouse IgG (H + L)-HRP**	Cell Signaling	#7076	Mouse Secondary	1:2000-10000	WB

Abbreviations: IHC, immunohistochemistry; WB, western blot; Aβ, amyloid β; FL-APP, full length amyloid precursor protein; Iba1, ionized calcium binding adaptor molecule 1; NeuN, neuronal nuclei; neuronal marker; GluR1, glutamate receptor 1; GluR2, glutamate receptor 2; PSD95, post-synaptic density protein 95

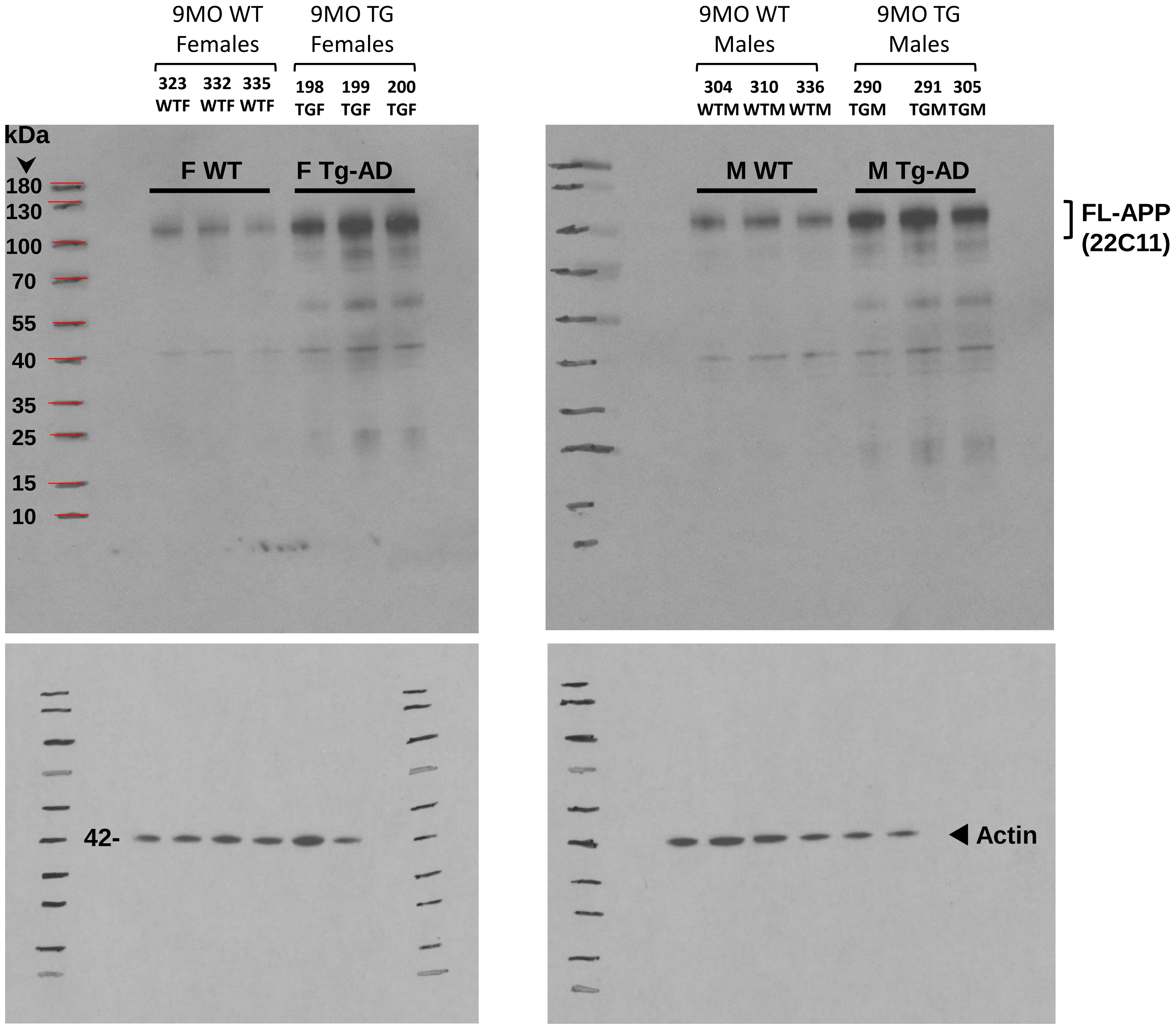

Uncropped blot for detection of FL APP and Actin in 9-month Female and Male WT and Tg-AD rats as shown in Fig. 6.
The numbers refer to specific rats.

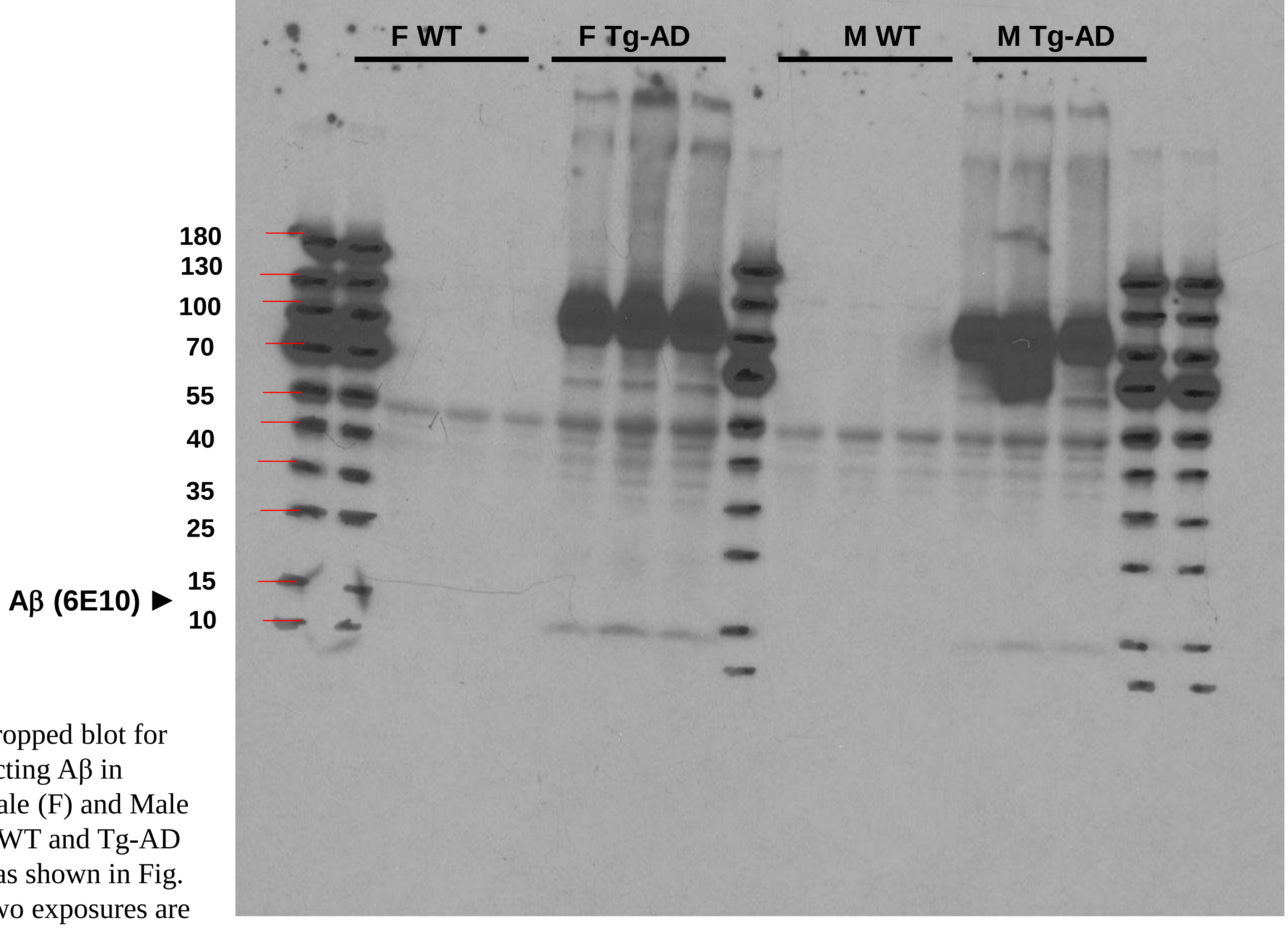

Uncropped blot for detecting Aβ in Female (F) and Male (M) WT and Tg-AD rats as shown in Fig. 6. Two exposures are shown.

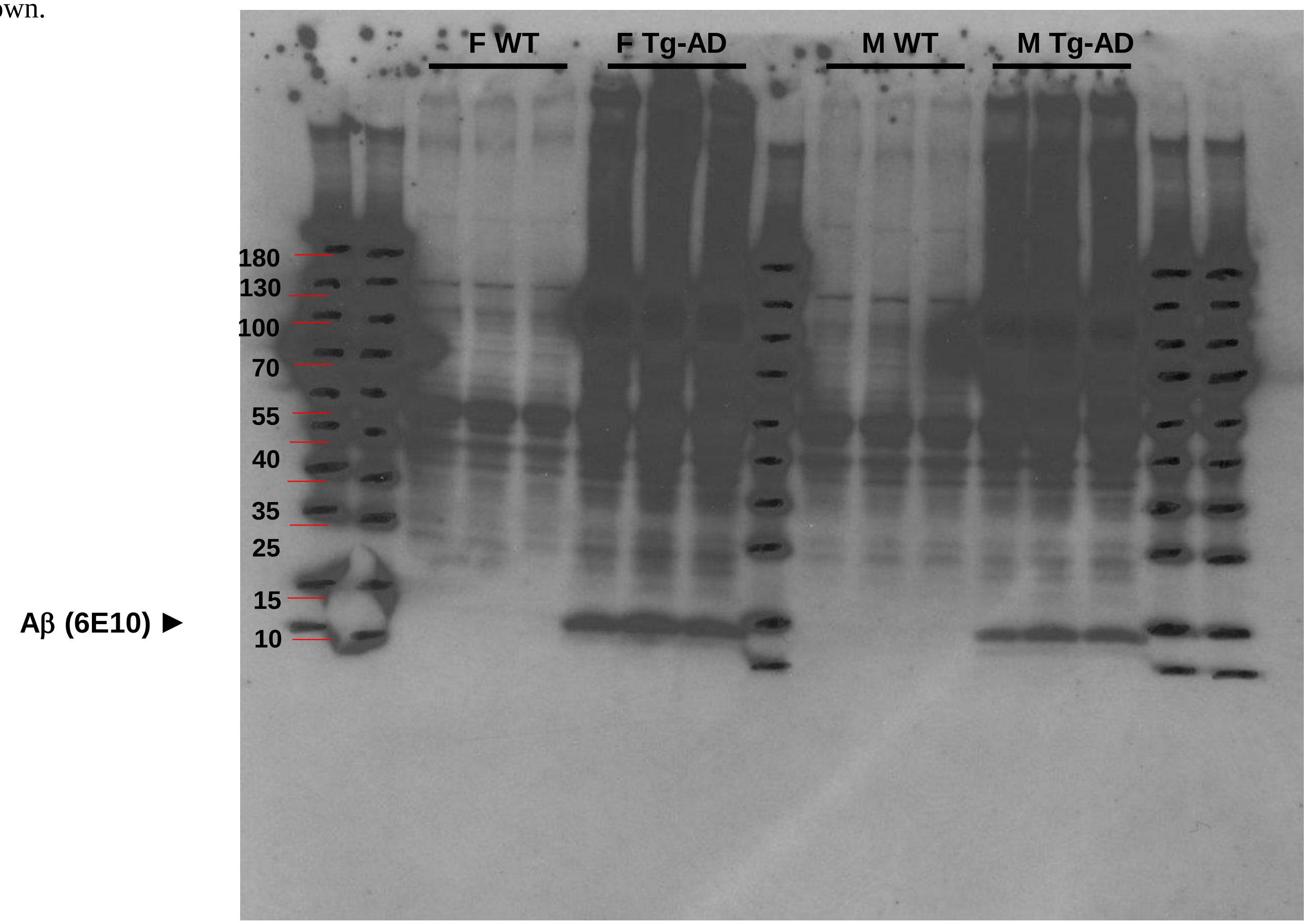

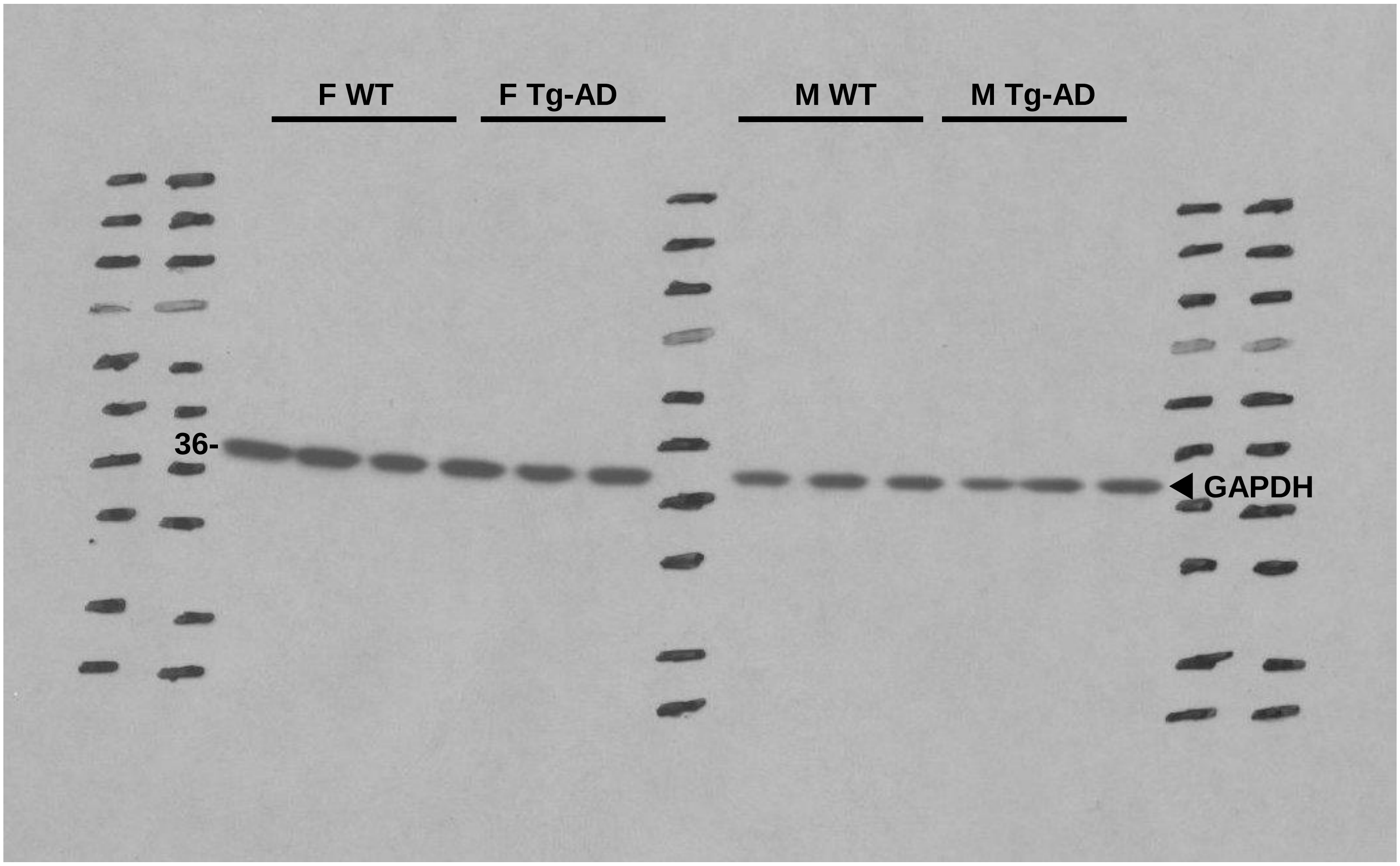

Uncropped blot for detection of GAPDH in Female (F) and Male (M) WT and Tg-AD rats as shown in Fig. 6.
